# Expanding the phenotypic spectrum of ILNEB: Absence of classic dermatologic findings in a 12-year-old girl with homozygous ITGA3 p.Arg274Gln

**DOI:** 10.1016/j.jdcr.2026.05.017

**Published:** 2026-05-14

**Authors:** Rahaf Bashihab, Alanoud Sultan Alsuhibani, Aser Mohammed Alsuhaymi, Mohammed Abdulaziz Alosaimi

**Affiliations:** a Division of Dermatology, King Abdulaziz Medical City, Ministry of National Guard Health Affairs, Riyadh, Saudi Arabia; b King Saud Bin Abdulaziz University for Health Sciences, College of Medicine, Riyadh, Saudi Arabia

**Keywords:** epidermolysis bullosa, hypotrichosis, ILNEB, interstitial lung disease, ITGA3, renal hypoplasia

## Introduction

Interstitial lung disease, nephrotic syndrome, and epidermolysis bullosa (ILNEB) is a rare autosomal recessive disorder caused by pathogenic variants in ITGA3, which encodes the α3 subunit of integrin α3β1.^1^, ^2^, ^3^, ^4^ Reported patients usually have varying combinations of early interstitial lung disease, renal involvement, and skin fragility resembling junctional epidermolysis bullosa.^1^, ^2^, ^3^, ^4^ More recent reports suggest that the phenotype may be broader than was initially appreciated, with some patients showing milder or organ-predominant disease (Table I).^5^, ^6^, ^7^ We report a 12-year-old girl with genetically confirmed ILNEB who had progressive pulmonary disease and unilateral renal hypoplasia, but no classic cutaneous fragility.

**Table I:** ILNEB cases explicitly reported without skin involvement (any ITGA3 variant)

Year	Country	Age/Sex	Genotype	Lungs	Kidneys	Outcome/Notes
2012	Netherlands	Neonate → 7 mo	Homozygous p.Ala349Ser (gain-of-glycosylation)	Severe interstitial lung disease	Congenital nephrotic syndrome; unilateral kidney hypoplasia	No macroscopic skin abnormalities; died at 7 mo^5^
2025	Saudi Arabia	12 y F (this case)	Homozygous p.Arg274Gln	Recurrent hypoxic admissions; restrictive PFT; GGO/mosaic attenuation	Left kidney hypoplastic/nonfunctioning; normal creatinine; no proteinuria	No clinical EB; alive, on follow-up (this report)

*ILNEB*, Interstitial lung disease, nephrotic syndrome, and epidermolysis bullosa.

## Case report

A 12-year-old Saudi girl born to a consanguineous couple was referred for evaluation of progressive respiratory disease. She had been well until 8 years of age, when she started to have recurrent respiratory infections that required repeated hospitalizations. By 9 years of age, she had repeated upper and lower respiratory tract infections that gradually became more severe, leading to multiple admissions for parenteral antibiotics and noninvasive respiratory support. Her presentation was initially attributed to asthma with frequent exacerbations. At 10 years of age, her condition worsened further and required 2 prolonged pediatric intensive care unit admissions, after which she was transferred to a tertiary care center.

Pulmonary evaluation showed markedly reduced forced vital capacity and forced expiratory volume in 1 second, with minimal bronchodilator reversibility, consistent with restrictive lung disease. High-resolution computed tomography showed diffuse patchy ground-glass opacities, mosaic attenuation, and mild pleural thickening of the left upper lobe. Bronchoscopy, bronchoalveolar lavage, and right lung biopsy showed nonspecific inflammatory changes. Taken together, these findings favored interstitial lung disease. In children with progressive diffuse lung disease, genetic testing can be helpful in reaching a unifying diagnosis.^8^

During admission, nephrology was consulted after the family reported a previous incidental finding of a nonfunctioning left kidney. Renal ultrasonography showed a hypoplastic left kidney measuring 2.3 cm and a compensatorily enlarged right kidney measuring 13 cm, with preserved corticomedullary differentiation and no hydronephrosis or stones. Serum creatinine, blood urea nitrogen, and albumin were within normal limits, and urinalysis showed no proteinuria. She had no history of edema or recurrent urinary tract infections. Because renal function was preserved, annual follow-up was advised. Renal developmental anomalies have also been described with ITGA3 mutations.^9^

**Fig 1:**
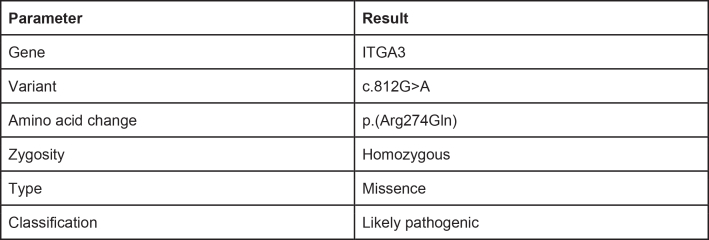
Whole exome sequencing result demonstrating a homozygous ITGA3 c.821G>A (p.Arg274Gln) variant, classified as likely pathogenic.

Whole exome sequencing identified a homozygous likely pathogenic variant in ITGA3, c.821G>A (p.Arg274Gln) (Fig 1), consistent with autosomal recessive junctional epidermolysis bullosa type 7 with interstitial lung disease and nephrotic syndrome, also known as ILNEB. The variant was classified according to ACMG/AMP criteria.^10^ This result provided a unifying explanation for her pulmonary and renal findings despite the absence of classic dermatologic disease.

**Fig 2:**
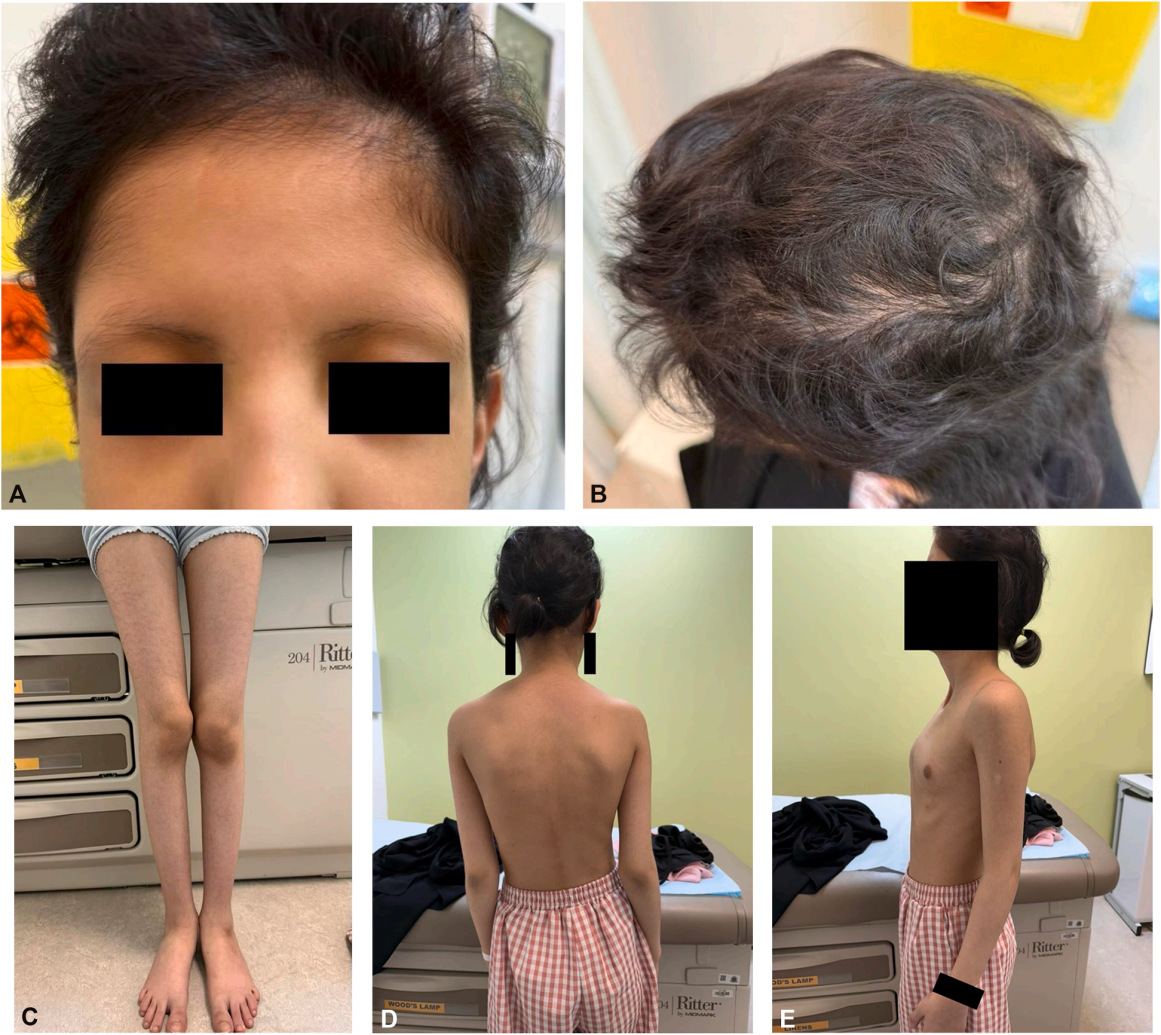
Clinical findings. **A,** Sparse eyebrows and frontal scalp thinning. **B,** Decreased scalp hair density consistent with hypotrichosis. **C,** Long lower limbs supporting marfanoid habitus. **D,** Scoliosis. **E,** Long upper limb supporting marfanoid habitus.

After the genetic diagnosis, she was evaluated in pediatric dermatology. Full examination of the skin, hair, nails, and mucosa showed no evidence of epidermolysis bullosa, and there was no history of blistering, erosions, or skin fragility. Nail and mucosal findings were likewise unremarkable. The main positive findings were congenital hypotrichosis and sparse eyebrows. She also had xerosis and marfanoid body habitus with scoliosis and disproportionately long upper and lower limbs. In addition, she had hirsutism and striae, which were thought to be related to prior systemic corticosteroid exposure. She was advised to start topical minoxidil 5% once daily, continue regular emollient use, and undergo hair loss labs. The family was counseled regarding home surveillance and was asked to photograph any future skin lesions (Fig 2).

## Discussion

This case is notable because the clinical picture did not match the classic triad usually expected in ILNEB (Table II). The patient had significant pulmonary disease and a structural renal abnormality, but she did not have the mechanobullous skin findings that are usually emphasized in ITGA3-related disease.^1^, ^2^, ^3^, ^4^ Her cutaneous findings were limited to hypotrichosis, sparse eyebrows, and xerosis.

**Table II:** Our case vs “classical” ILNEB vs attenuated phenotypes

Domain	Our case	“Classical” ILNEB (first reports)	Attenuated/viable ILNEB phenotypes
Age at presentation	Recurrent URTIs at 9-10 y; diagnosis at 12 y	Neonatal/infant onset; rapid decline	Childhood onset; survival beyond early years
Pulmonary	Restrictive PFTs; GGO + mosaic attenuation on HRCT; multiple admissions	Severe ILD early; high mortality	Variable ILD; some stabilize; occasional long-term survival
Renal	No proteinuria; structural anomaly (hypoplastic left kidney); normal renal function	Congenital nephrotic syndrome typical	Proteinuria may be absent; CAKUT/hypodysplasia reported
Dermatologic	No EB; xerosis; mild hypotrichosis; sparse eyebrows; hirsutism; striae	Skin fragility/EB common	Range from subtle adnexal findings to mild fragility
Genetics	ITGA3 c.821 G > A (p.Arg274Gln) homozygous	Biallelic ITGA3 variants	p.Arg274Gln previously linked to attenuated disease
Outcome/plan	Multidisciplinary stabilization; dermatologic surveillance; topical minoxidil 5%	Supportive care; high infant mortality	Organ-directed care; transplant in end-stage cases
References	—	^1^	^8^^,^^9^

*ILNEB*, Interstitial lung disease, nephrotic syndrome, and epidermolysis bullosa.

The overall course also appears milder than that described in many early reports. She survived into later childhood, had preserved renal function, and never developed proteinuria, despite having a confirmed homozygous ITGA3 variant.^5^^,^^6^ Similar survival and phenotypic variability have been described, but they remain relatively uncommon.^5^, ^6^, ^7^

Another important point is the diagnostic course. For a prolonged period, her respiratory symptoms were managed as asthma with frequent exacerbations. In retrospect, the poor bronchodilator response, restrictive pulmonary function pattern, and abnormal high-resolution computed tomography findings were more suggestive of childhood interstitial lung disease.^8^ This case highlights the importance of reconsidering the diagnosis when a presumed asthma picture becomes unusually severe, recurrent, or poorly responsive to standard therapy (Table III).

**Table III:** Summary of published ITGA3 p.Arg274Gln (R274Q) cases

Year	Country	Age/Sex	Genotype	Phenotype summary	Organ involvement	Outcome/Notes
2016	Italy	13y, 9y (siblings)	Compound het: p.Gly125Arg + p.Arg274Gln	Pulmonary fibrosis; sparse eyebrows/lashes; nail dystrophy	Lung+, kidney (no nephrotic impairment), Skin (mild)	Alive at report; p.Arg274Gln suggested hypomorphic^8^
2023-2024	Saudi Arabia	15 y F, 13 y M, 2y F (siblings)	Homozygous p.Arg274Gln	JEB variant with hypotrichosis, nail dystrophy, lacrimal obstruction; no renal/respiratory disease	Skin/hair/mucosa; Lungs−; Kidneys−	Prolonged survival without renal/respiratory symptoms^9^
2025	Saudi Arabia	12 y F (this case)	Homozygous p.Arg274Gln	Respiratory-predominant; dermatology: xerosis, mild hypotrichosis only	Lung+; kidney (left hypoplastic, normal function); Skin− (no EB)	Ongoing multidisciplinary care (this report)

In summary, we report a 12-y-old girl with homozygous ITGA3 p.Arg274Gln-associated ILNEB presenting with interstitial lung disease, unilateral renal hypoplasia, and no classic epidermolysis bullosa. This case broadens the recognized clinical spectrum of ILNEB and shows that absence of the expected dermatologic findings may delay recognition of the diagnosis.

Integrin α3β1 plays an important role in epithelial stability in the lungs, kidneys, and skin.^1^, ^2^, ^3^, ^4^ Variable disruption of this pathway may explain why some patients show the full triad, whereas others have lung-predominant disease, limited cutaneous findings, or milder renal involvement. Our patient supports the view that ILNEB should still be considered even when blistering is absent, especially if pulmonary disease and renal abnormalities coexist.

## Conflicts of interest

None disclosed.
